# The Complex Genetic Context of *bla*_PER-1_ Flanked by Miniature Inverted-Repeat Transposable Elements in *Acinetobacter johnsonii*

**DOI:** 10.1371/journal.pone.0090046

**Published:** 2014-02-25

**Authors:** Zhiyong Zong

**Affiliations:** 1 Center of Infectious Diseases, West China Hospital, Sichuan University, Chengdu, Sichuan Province, China; 2 Division of Infectious Diseases, The State Key Laboratory of Biotherapy, Sichuan University, Chengdu, Sichuan Province, China; Universidad Nacional de La Plata., Argentina

## Abstract

On a large plasmid of *Acinetobacter johnsonii* strain XBB1 from hospital sewage, *bla*_PER-1_ and IS*CR1* were found in a complex Tn*402*-like integron carrying an *arr3*-*aacA4* cassette array. The integron was truncated by the same 439-bp miniature inverted-repeat transposable element (MITE) at both ends. *bla*_PER-1_ and its complex surroundings might have been mobilized by the MITEst into an orf of unknown function, evidenced by the presence of the characteristic 5-bp direct target repeats. The same 439-bp MITEs have also been found flanking class 1 integrons carrying metallo-β-lactamases genes *bla*_IMP-1_, *bla*_IMP-5_ and *bla*_VIM-2_ before but without IS*CR1*. Although the cassette arrays are different, integrons have always been truncated by the 439-bp MITEs at the exact same locations. The results suggested that MITEs might be able to mobilize class 1 integrons via transposition or homologous recombination and therefore represent a possible common mechanism for mobilizing antimicrobial resistance determinants.

## Introduction

*bla*_PER-1_ encodes the extended-spectrum β-lactamase (ESBL) PER-1 conferring resistance to penicillins, cephalosporins and monobactams [1] and has been found in *Aeromonas* spp., *Acinetobacter baumannii*, *Alcaligenes faecalis*, *Pseudomonas aeruginosa* and the *Enterobacteriaceae* in Asia and Europe [2], [3]. The production of PER-1 by Gram-negative bacilli of clinical significance compromises the option for antimicrobial chemotherapy. It has been well established that the mobilization of antimicrobial resistance determinants such as ESBL-encoding genes is largely mediated by mobile genetic elements including insertion sequences, transposons, integrons and gene cassettes. Recently, miniature inverted-repeat transposable elements (MITEs), which are small non-autonomous mobile elements containing repeated sequences and are present in diverse bacteria [4], have also been found mediating the mobilization of antimicrobial resistance determinants [5]. *bla*_PER-1_ has been found in diverse genetic contexts and is generally located downstream of the insertion sequence IS*Pa12* [2]. However, *bla*_PER-1_ had not been found associated with MITEs before [2].

*Acinetobacter johnsonii* (*Acinetobacter* genospecies 7) is a bacterial species that has usually been found in the aquatic environment [6] but occasionally colonizes humans [7] or causes clinical infections [8]. A strain of *A. johnsonii* was recovered from hospital sewage in western China and was found carrying *bla*_PER-1_. Here the genetic context of *bla*_PER-1_ was examined in detail.

## Materials and Methods

### Strain and the Detection of *bla*_PER_

*A. johnsonii* XBB1 was obtained from sewage collected at the influx of the wastewater treatment plant in West China Hospital, Chengdu, China, on October 2010 and was identified as *A. johnsonii* by partially sequencing the 16S rRNA gene [9]. No specific permissions were required for the activities to obtain the sewage as the wastewater treatment plant is a closed unit in the hospital and the field studies did not involve endangered or protected species. *A. johnsonii* XBB1 was resistant to ceftriaxone and ceftazidime [9] and the presence of *bla*_PER_ gene was detected using PCR with self-designed primers (PER-UF, 5′-CCTGACGATCTGGAACCTTT; PER-UR, 5′-TCATCGASGTCCAGTTTTGA). PCR was performed using ExTaq premix (Takara, Dalian, China) and the following conditions: 94°C for 5 min; 30 cycles of 94°C for 30 s, 55°C for 30 s, 72°C for 1 min; and a final elongation step at 72°C for 7 min.

### Genome Sequencing and Assembly

*A. johnsonii* XBB1 was subjected to the whole genome sequencing. The genomic DNA of XBB1 was prepared using the QIAamp DNA Mini kit (Qiagen, Hilden, Germany) and was then sequenced using the Roche 454 GS FLX+ (Roche 454 Life Sciences, Branford, CT, USA) and the Illumina Paired-end (Illumina, San Diego, CA, USA) platforms at the Beijing Genomics Institute (Beijing, China). Reads containing at least 50-bp non-adapter non-primer sequence generated by the 454 platform were assembled using the Newbler program (version 2.6) using 40 bp as the minimum overlap length and 90% as the minimum overlap identity. The assembled sequences were further assembled with reads generated by the Illumina platform using the Sspace program (version 2.0). The sequence gap of the plasmid carrying *bla*_PER-1_ was filled in using PCR with primers designed based on sequences available and Sanger sequencing using an ABI 3730×l DNA Analyzer (Applied Biosystems, Foster City, CA, USA). Similarity searches of sequences obtained were carried out using BLAST programs (http://www.ncbi.nlm.nih.gov/BLAST/) and the function of proteins was predicted using the InterProScan (http://www.ebi.ac.uk/Tools/pfa/iprscan/).

### Verification of the Genetic Context of *bla*_PER-1_

**Figure 1:**
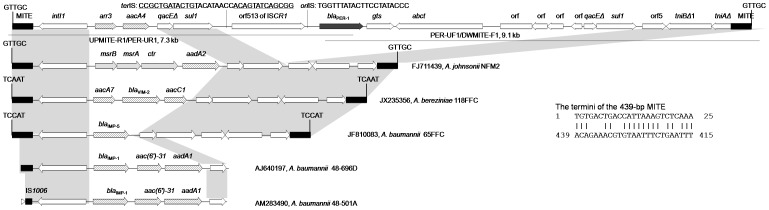
Structures flanked by the 439-bp MITE. Two overlapping long-range PCR are shown by lines with primer names and amplicon sizes being indicated. The MITEs shown here have identical nucleotide sequences and are in the same direction. The 5-bp DR characteristics of the transposition of MITE-form composite transposon-like element are indicated. The GenBank accession numbers, the host species and strain number of each structure are listed. The identical regions are highlighted in grey. Only partial sequences are available for the integron carrying *bla*_IMP-1_ (GenBank accession numbers AM283490 and AJ640197). The *ori*IS and the putative *ter*IS of IS*CR1* are shown and the inverted repeats of *ter*IS [23] are underlined. The 25-bp terminal sequences of this 439-bp MITE are shown with 18 bp matched.

To confirm the genetic context of *bla*_PER-1_, two overlapping long-range PCR were employed with primer pair PER-UF/DWMITE-F1 and PER-UR/UPMITE-R1. Self-designed primers DWMITE-F1 (5′-TGGCTCAATGTCTGATTGCT) and UPMITE-R1 (5′-TTGTTTGGGATTTGGTCCTC) were located 141 and 166 bp away from each end of the 15.3 kb large region containing *bla*_PER-1_, respectively (Figure 1). The conditions of long-range PCR (Fermentas, Burlington, ON, Canada) were 94°C for 2 min; 10 cycles of 94°C for 10 s, 55°C for 30 s, 68°C for 12 min; then 25 cycles of 94°C for 15 s, 55°C for 30 s, 68°C for 6 to 12 min plus 10 s cycle elongation for each successive cycle; and a final elongation step at 68°C for 7 min. The amplicons were sequenced at both directions (see above).

#### Nucleotide sequences accession number

Sequence of the genetic context of *bla*_PER-1_ in *A. johnsonii* XBB1 has been deposited in GenBank as KF017283.

## Results and Discussion

The genetic region containing *bla*_PER-1_ in XBB1 generated by assembling whole genome sequencing reads was verified by the overlapping long-range PCR and Sanger sequencing. In XBB1, *bla*_PER-1_ was carried by a 399 kb large plasmid, which had been completely sequenced, assembled and circularized. *bla*_PER-1_ was found downstream of the insertion sequence IS*CR1* in a 5.7 kb region that included *gts* (encoding a glutathione *S*-transferase), *abct* (encoding an ABC-type transporter) and four genes of unknown function in a complex Tn*402*-like class 1 integron with the *arr3* and *aacA4* cassettes (Figure 1). The IS*CR1*-*bla*_PER-1_ structure was bracketed by two copies of 3′ conserved segment (3′CS), which comprised a truncated *qacE* gene encoding resistance to quaternary ammonium compounds and the sulphonamide resistance gene *sul1*. The 5′ conserved segment (5′CS) and the Tn*402*-like *tni* transposition module (*tniBΔ1*-*tniA*) were truncated by the same 439-bp MITE) (Figure 1). The two MITEs, which were in the same direction, might have formed a composite transposon-like mobile element mediating the transposition of the complex Tn*402*-like class 1 integron containing IS*CR1* and *bla*_PER-1_ into a gene of unknown function as evidenced by the presence of characteristic 5 bp (GTTGC) direct target repeats (DR, Figure 1).

This MITE is AT-rich (AT content, 72.2%), has no orf and possesses terminal sequences without significant matches to inverted repeats of any known insertion sequences and transposons. The same (100% nucleotide identity) 439-bp MITEs have also been found flanking Tn*402*-like class 1 integrons carrying different cassette arrays but without IS*CR1* in several *Acinetobacter* strains (Figure 1). In *A. johnsonii* strain NFM2 that was recovered from a prawn in Australia [10], the 439-bp MITEs flanked an unusual class 1 integron that carried methionine sulfoxide reductase genes and this MITE-flanked element was inserted into a location exactly the same as that seen in the present study. In *A. baumannii* clinical strain 65FFC, MITEs flanked a class 1 integron containing a single *bla*_IMP-5_ cassette and this MITE-flanked structure was transposed into the transposase-encoding *tnpA* gene of an IS*Aba14*-like element, generating 5-bp (TCCAT) DR [5]. In *Acinetobacter bereziniae* strain 118FFC (GenBank accession number JX235356), a class 1 integron with the *aacA7*-*bla*_VIM-2_-*aacC1* cassette array was flanked by MITEs, which were mobilized into the transposase-encoding *tnpA* gene of IS*26* evidenced by the presence of 5-bp (TCAAT) DR (Figure 1). Partial MITEs have also been found to truncate the 5′CS of a class 1 integron carrying the *bla*_IMP-1_-*aac(6*′*)-31-aadA1* cassette array in *A. baumannii* strain 48–501A (AM283490) and strain 48–696D (AJ640197). Only partial MITE sequence is provided for strain 48–696D, while MITE is truncated by IS*1006* in strain 48–501A. No sequences downstream of 3′CS are available for both strains and therefore it remains unknown whether there is another copy of MITE at the other end of In86. *bla*_IMP-1_, *bla*_IMP-5_ and *bla*_VIM-2_ are genes encoding class B metallo-β-lactamases. Of note, the MITE-flanked structures containing Tn*402*-like integrons have been seen interrupting different genes with the presence of DR, suggesting that the MITEs indeed mediated the mobilization of integrons. Interestingly, MITE always truncated the 5′CS and *tniA* at the same locations in all known sequences regardless of the cassette array and host species or strains, suggesting that the truncation of Tn*402*-like integrons by MITEs at either ends might have only occurred once and the different cassette arrays might therefore have resulted from homologous recombination between integrons. The association of MITEs with a complex class 1 integron containing IS*CR1* was not seen before and the IS*CR1*-*bla*_PER-1_ region seen in the present study might have also been introduced by homologous recombination with the 5′CS or 3′CS serving as one homologous region and the *tni* module serving as the other required for double crossover. There is also evidence that circular molecules created by recombination in the duplicated 3′CS flanking regions containing IS*CR*-antimicrobial resistance gene [11]. Although the MITEs-flanked structures seen in strain NFM2 from Australia and *A. johnsonii* XBB1 from China had different genetic components, they were present at the same location, suggesting that the acquisition of the IS*CR1*-*bla*_PER-1_ region might have occurred later than the transposition mediated by MITEs. As mobile genetic elements, MITEs might be able to mobilize *bla*_PER-1_ into different plasmids within the host strain and then could generate various genetic scaffolds to facilitate the horizontal transfer of the ESBL gene between clinical isolates and those of an environmental origin. The presence of *bla*_PER-1_ within the mobile genetic element formed by MITEs from an environment isolate in a hospital setting is therefore of significance.

To date, *bla*_PER-1_ has been found in genetic contexts either associated with the insertion sequence IS*Pa12* or with IS*CR1* (Table 1). IS*Pa12* and its close relative IS*Pa13* formed a composite transposon termed Tn*1213*, which realized the mobilization of *bla*_PER-1_ and a truncated *gts* remnant as evidenced by the presence of characteristic 8-bp DR. Several variants of Tn*1213* have also been identified (Table 1), including IS*6100* or IS*Ppu17* truncating IS*Pa12* and IS*Prst1* inserting into Tn*1213* [2], [12], [13]. Tn*1213* has been found in various species in Europe and Asia and therefore appears to be a common mechanism mediating the mobilization of *bla*_PER-1_. In particular, Tn*1213* and its variants were the only type genetic context of *bla*_PER-1_ identified in *P. aeruginosa* so far. Another type of association of IS*Pa12* with *bla*_PER-1_ has been seen in *A. baumannii* strain C.A. and *Salmonella enterica*, in which the insertion of IS*Pa12* at 57 bp upstream of *bla*_PER-1_ was independently of the *bla*_PER-1_ acquisition as the presence of the characteristic 8-bp DR flanking IS*Pa12* suggested transposition of IS*Pa12* on its own [2]. In these cases, the mechanisms for the mobilization of *bla*_PER-1_ remain undetermined. The association of IS*CR1* with *bla*_PER-1_ has been found in two *A. baumannii* isolates (Table 1) before but no further sequence data were available to identify the integrons in which IS*CR1* and *bla*_PER-1_ might be embedded and to demonstrate whether MITEs were also involved like the case seen in the present study. Of note, there are two spacer sizes between IS*CR1* and *bla*_PER-1_, suggesting that the acquisition of *bla*_PER-1_ by IS*CR1* might have occurred more than once. As the spacer between IS*CR1* and *bla*_PER-1_ was longer than that between IS*Pa12* and *bla*_PER-1_ and *gts* was truncated in Tn*1213*, it might be reasonable to propose that the acquisition of *bla*_PER-1_ by IS*Pa12* is a more recent event when compared to the acquisition by IS*CR1*. Both Tn*1213* and the IS*CR1*-*bla*_PER-1_ context have been identified in *A. baumannii*, suggesting that *A. baumannii* might be the host species in which different platforms for mobilizing *bla*_PER-1_ had been formed.

**Table 1:** Genetic contexts of *bla*_PER-1_ *^a.^*

Genetic context^b^	Species and strain	Country	IS-*bla*_PER-1_spacer (bp)	Accessionno.	Reference
**IS** ***Pa12*** **-IS** ***Pa13*** ** composite transposon** c					
IS*Pa12*-*bla*_PER-1_-*gts*Δ-IS*Pa13*	*A. baumannii* AMA-1	France	13		[2], [14]
	*A. baumannii* 1656-2	Korea	13	CP001921	[15]
	*A. baumannii* strains 7 and 8	Belgium	13		[16]
	*Aeromonas media* A72	Switzerland	13		[3]
	*Klebsiella pneumoniae* CS1711	Korea	13		[17]
	*P. aeruginosa* RNL-1	France	13	AY779042	[2], [18]
	*P. aeruginosa* MUL	France	13		[2]
	*P. aeruginosa* 1	Turkey	13		[2]
	*P. aeruginosa* PER12	Belgium	13		[2], [19]
	*P. aeruginosa* 2622	Poland	13		[2]
	*P. aeruginosa* PA2345	France	13	AY866517	[20]
IS*Pa12*-*bla*_PER-1_-*gts*Δ-IS*Prst1*-*gts*Δ-IS*Pa13*	*Providencia stuartii* BEN	France	13		[2]
IS*6100-* IS*Pa12*Δ-*bla*_PER-1_-*gts*Δ-IS*Pa13*	*P. aeruginosa* P66	Greece	13	FR847979	[12]
IS*Ppu17-* IS*Pa12*Δ-*bla*_PER-1_-*gts*Δ-IS*Pa13*	*Alcaligenes faecalis* FL-424/98	Italy	13	AJ627643	[13]
**IS** ***Pa12*** **-** ***bla*** **_PER-1_-** ***gts*** **-** ***abct*** **-?**	*A. baumannii* C.A.	Turkey	57		[2], [14]
	Serovar Typhimurium TUR	Turkey	57		[2], [21]
	Serovar Typhimurium 147	Turkey	57		[2]
**IS** ***CR1*** **-** ***bla*** **_PER-1_**					
MITE-*intI1*-*arr3*-*aacA4*-3′CS-IS*CR1*-*bla*_PER-1-_ *gts-* *abct*-4orfs-3′CS-orf5-*tniB*Δ-*tniA*Δ-MITE	*A. johnsonii* XBB1	China	80		This study
?-IS*CR1*-*bla*_PER-1_-*gts-abct*-?	*A. baumannii* NF161710	China	72	JQ780836	
	*A. baumannii* NF812784	China	80	GU944725	[22]

a The *bla*_PER_ gene in *Aeromonas punctata* strain 169 (GenBank accession number GQ871757) has also been annotated as *bla*_PER-1_ but it has two nucleotide differences from *bla*_PER-1_ (Z21957), specifying one amino acid substitution compared to PER-1. Therefore, this *bla*_PER_ gene was not included.

b ?, sequence remains unknown.

c IS*Pa12* is called as IS*1387a* in Tn*5393d* and IS*Pa23* in *P. aeruginosa* PA2345. IS*Ppu17* is annotated as IS*1066* in Tn*5393d* and IS*Pa13* is termed as IS*Pa24* in *P. aeruginosa* PA2345.

## Conclusion

A unique MITE-flanked complex Tn*402*-like integron carrying IS*CR1* and *bla*_PER-1_ was revealed in an *A. johnsonii* strain from hospital sewage. Such a complex integron flanked by a pair of 439-bp MITEs has never been reported before. The 439-bp MITEs might be able to mobilize class 1 integrons via direct transposition or homologous recombination. It appears that MITEs could serve as a common mechanism mediating the mobilization of antimicrobial resistance genes. This study has prompted an additional survey for the presence of MITEs in clinical isolates of *Acinetobacter* spp.
